# Data to Action: A Mixed-Methods Study of Data Use Teams, Improved Availability of Contraceptives in Guinea, Indonesia, Kenya, and Myanmar

**DOI:** 10.9745/GHSP-D-21-00345

**Published:** 2022-06-29

**Authors:** Ann-Marie Yongho, Yasmin Chandani, Sarah Andersson, Ali Karim, Bethany Saad, Carmit Keddem

**Affiliations:** a Independent, Washington DC, USA.; b InSupply Health, Nairobi, Kenya.; c Independent consultant, Melbourne, Australia.; d Bill & Melinda Gates Foundation, Seattle, WA, USA.; e John Snow, Inc., Boston, MA, USA.

## Abstract

Information Mobilized for Performance Analysis and Continuous Transformation (IMPACT) Teams routinely bring together data, people, processes, and technology, under the leadership of governments, to institute a change in culture that leads to sustained improvements in supply chain processes and outcomes. This mixed methods study examined whether IMPACT Teams were effective in improving reproductive health supply chain outcomes in Guinea, Indonesia, Kenya, and Myanmar and identified enablers and barriers to IMPACT Team success and sustainability in Indonesia and Kenya.

The study design employed a pre-post intervention comparison panel design with a nonrandomized matched comparison group to examine the IMPACT Teams' effect on 2 supply chain outcomes: stocked according to plan and stock-outs. Additional key informant interviews conducted in Kenya and Indonesia explored enablers and barriers to IMPACT Team success and sustainability.

For nearly all products across the 4 countries, an increase in products being stocked according to plan and a reduction in stock-outs can be attributed to the IMPACT Team intervention, demonstrating that IMPACT teams are an effective approach for improving contraceptive supply chain inventory management and availability. However, our findings do not demonstrate a clear causal pathway as theorized in our theory of change, namely that government leadership leads to the installation of a data use culture, which in turn leads to improved product availability. In both Indonesia and Kenya, though product availability improved, there was a lack of leadership and culture change. This suggests that improved product availability does not depend on establishing a data use culture or government leadership, but rather, that a data use culture—rather than product availability—is the outcome of interest for sustained change, and that understanding motivations and incentives for leadership participation may be more important for scaling, institutionalizing, and sustaining gains in supply chain outcomes.

## Abstract (precis)

Information Mobilized for Performance Analysis and Continuous Transformation (IMPACT) Teams use a people-centered, data-driven approach to strengthen supply chains by fostering a continuous cycle of supply chain improvement. This study demonstrates that IMPACT Teams are an effective approach for improving contraceptive supply chain inventory management and availability.

## Teaser

**Key Findings:**

- Information Mobilized for Performance Analysis and Continuous Transformation (IMPACT) teams are an effective strategy for improving reproductive health supply chain outcomes in the short term but can only be sustained through ownership and engagement by leaders.
- Although IMPACT Team interventions in 4 countries led to improvements in the supply chain, there was limited establishment of the data use culture.
- Lack of engagement by higher-level government entities—and absence of financial support for the IMPACT Team process—comprised major barriers to sustainability of the IMPACT Team approach.

**Key Implications:**

- The core components of data, technology, processes, and people, which are rooted in the quality improvement approach, need to be accompanied by intentional efforts to foster government ownership and leadership, so that this process is not only instilled but sustained over time.

Résumé en français à la fin de l'article.

## INTRODUCTION

Ensuring access to family planning has long been recognized as a key component to unlocking general health improvements in lower- and middle-income countries (LMICs), with benefits that include improving women's and children's health and reducing maternal and infant mortality.^1^^–^^6^ Yet, as recently as 2022, 9.2% of women in LMICs who wanted to avoid pregnancy had an unmet need for contraception, and the global rate of unmet need for modern contraception is 13.3%.^7^

One of the key barriers to ensuring a well-functioning family planning program and meeting unmet need is reliable access to contraceptive supplies.^3^^,^^8^^–^^11^ Stock-outs are common and pervasive in LMICs.^3^^,^^9^^,^^10^^,^^12^^–^^14^ Beyond unwanted or unplanned pregnancies, Zuniga et al. found that such stock-outs have many consequences for women, limiting their ability to use a preferred method, access to locations where the preferred method could be obtained, providers' ability to provide proper contraceptive care, and the cost for contraception.^15^ Furthermore, family planning providers reported that stock-outs of some methods negatively affect adherence when women are forced to switch methods and that repeated stock-outs can result in a loss of trust in the entire health facility.^9^

Recent improvements in supply chains focusing on data availability and use have seen promising results.^14^^,^^16^^,^^17^ When studying the implementation of the Informed Push Model in Senegal, Daff et al. found that having access to timely and accurate consumption data allowed district and regional managers to quickly identify and address performance issues.^14^ They also found that eliminating stock-outs and thereby increasing method choice had knock-on effects in providers being more likely to advise clients on the full range of methods available, increasing the overall use of family planning.^14^ This complements other research findings suggesting that providing more options results in increased use of contraceptives.^3^^,^^10^

Chandani et al. found that interventions to strengthen community health supply chains work best when they address 3 key areas: product flow, data flow, and effective people.^17^ In other words, the correct data needs to flow to the correct person or people in the supply chain, who must then have the skills and authority to analyze the data, make key decisions, and ensure product flow.^17^ Furthermore, their results suggest that teams composed of representatives from multiple levels of the health system are more effective in solving supply problems than single-level teams or authorities.^17^ These observations are reinforced by industry best practices which demonstrate that the ongoing collaboration and integration of actors along the supply chain supports aligned objectives and information sharing and improves overall LMIC supply chain management and performance.^18^ The authors should place these observations in this wider context as an opportunity to endorse the adoption of industry practice in LMIC health supply chains. This is in line with common industry best practices These findings complement Daff et al.'s conclusion that having access to timely and accurate data in Senegal fostered a growing culture of data-driven performance improvement throughout the health system, beyond the family planning sector.^14^

We examined an intervention implemented in multiple countries that seeks to achieve targeted gains in contraceptive supply chain performance through improved data visibility and use by multilevel decision-making teams, called Information Mobilized for Performance Analysis and Continuous Transformation (IMPACT) Teams (Box 1).^19^

BOX 1 **What Are IMPACT Teams?**

**FIGURE 1:**
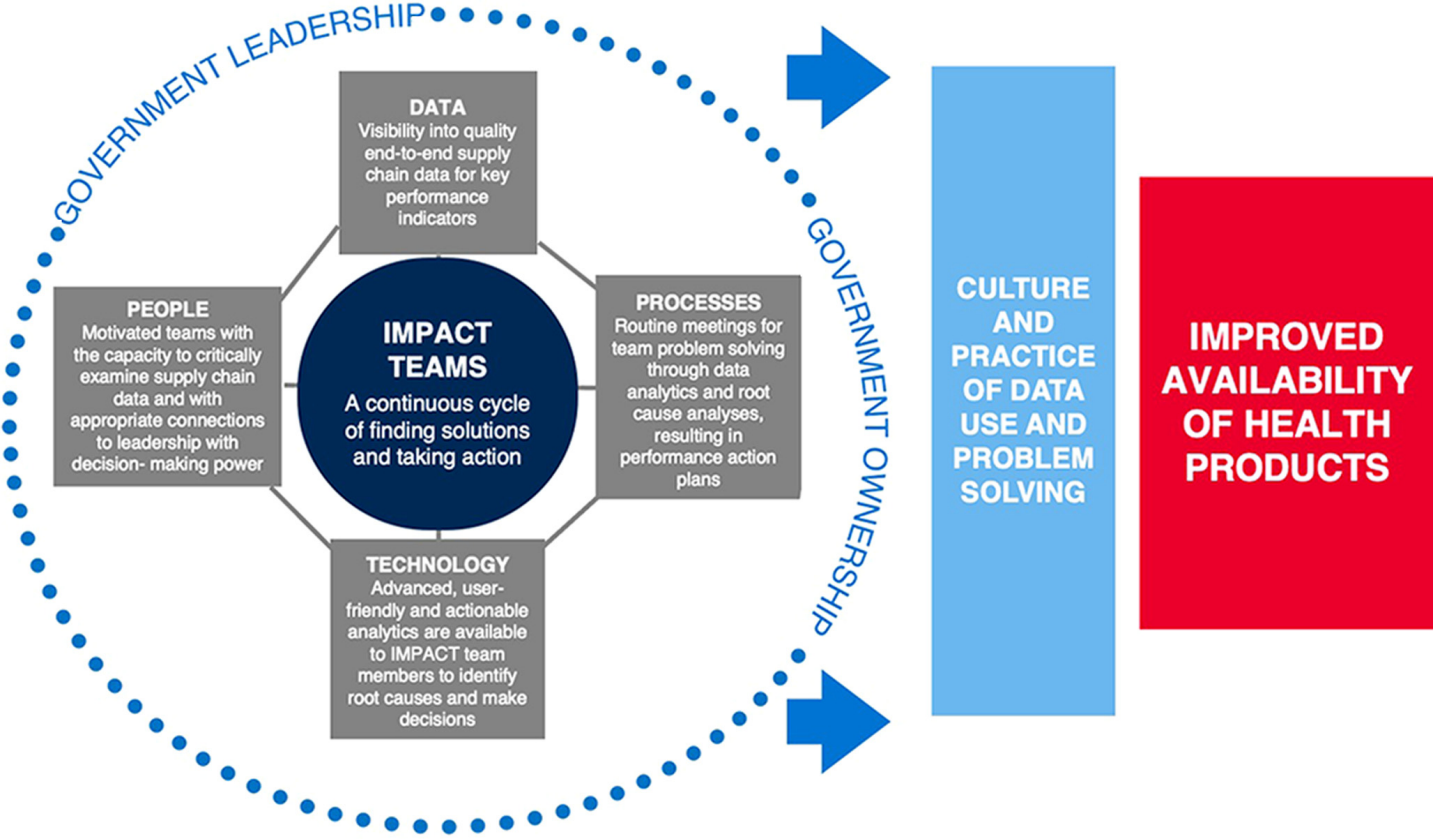
IMPACT Team Theory of Change Abbreviation: IMPACT, Information Mobilized for Performance Analysis and Continuous Transformation.

Information Mobilized for Performance Analysis and Continuous Transformation (IMPACT) Teams evolved from modern quality improvement strategies adopted from private-sector industry to create a people-centered, data-driven approach to strengthening supply chains.^21^ The IMPACT Team intervention was designed to foster a continuous cycle of supply chain improvement. The approach brings together key supply chain data, appropriate technology, and people working across functions and disciplines through a structured process—under the leadership of local and subnational governments—to institute a change in culture that leads to sustained improvements in supply chain processes and outcomes. To understand and study the pathway to change, we developed a theory of change (TOC) (Figure 1), which highlights the following 4 core components.

**1. Data:** IMPACT Teams rely on strong, timely, and visible data systems to generate analyses that are usable, actionable, and relevant. IMPACT Teams identify and regularly track key performance indicators that reflect the data available as well as the programmatic scope of each team.

**2. Technology:** Real-time data about supplies has become more visible and available as countries have invested in building electronic and automated logistics management information systems (LMIS) and training staff to report into these systems. IMPACT Teams use various technological solutions to create user-friendly, actionable dashboards to guide the continuous review cycle.

**3. Processes:** Data availability and electronic LMISs alone, however, do not translate into data use and action. Therefore, teams are trained to follow specific processes: specifically, meeting regularly to critically examine and collectively interpret supply chain data that are presented using standardized indicators and user-friendly decision-support tools or dashboards. After reviewing the data, teams conduct a root cause analysis, prioritize problems, and create an action plan with solutions specific to local bottlenecks. Following each meeting, action plans are shared up and down the supply chain for action. Teams should be implemented at various levels of the supply chain, with health systems leaders actively participating in the team.

**4. People**: This process would not be possible without motivated team members who offer a mix of skills including data analysis and interpretation, supply chain management, and leadership, as well as the authority to translate identified solutions into concrete action. National and subnational stakeholders participate in capacity-building activities on data analysis and data literacy skills. Also, to foster motivation, recognition of individual performance is a key component of the IMPACT Team approach.

Since 2010, IMPACT Teams have been implemented across programs and health system levels in 10 countries. The SC4CCM Project adopted the quality collaboratives approach for health supply chains to improve knowledge and practices among community health workers by implementing multilevel quality improvement teams (QITs) in Malawi, Rwanda, and Ethiopia from 2010 to 2014.^20^ A key finding of this project was that QITs could offer an effective way to improve supply chain practices and performance but might need to be simplified and adapted for different contexts to ensure long-term ownership and continuity.^20^

Building on this evidence, between 2015 and 2019, Guinea, Indonesia, Kenya, and Myanmar implemented IMPACT Teams—simplified and modified from the original QITs to incorporate leadership elements—for their product health supply chains hypothesizing that contextual and program adaptations could achieve positive gains for supply chains across all levels and health programs.

IMPACT Teams were a central component of each implementation package (Table 1). While each instance was contextualized to the host country, the core components of IMPACT Teams remained the same. Both Indonesia and Myanmar carried out additional activities that accompanied IMPACT Teams.

**TABLE 1.** Overview of IMPACT Team Approach in Guinea, Indonesia, Kenya, and Myanmar

	Guinea	Indonesia	Kenya	Myanmar
Description of full package of interventions	IMPACT Teams			
		Strengthening supply chain inventory management procedures and records On-the-job training and supportive supervision		Establishment of the Reproductive Health Commodity Logistics System
Intended level of IMPACT Team implementation	Central and district	Province and district	County (included subcounty staff on demand)	Township and health facility; state level in last 2 years of project
Frequency of IMPACT Team meetings	Monthly	Monthly	Monthly	Monthly; state level quarterly
Project support provided to IMPACT Teams	Financial, technical support for design, training of the teams			
	Technical advisor to support and participate, capacity building of teams in data use processes^a^			
	Mid-project meeting, refresher training	Meeting cost support for multilevel (province with district) meetings held outside the office	Meeting cost support for multilevel (county, subcounty) meetings held outside the office	No financial or logistical support was provided for routine IMPACT Team meetings
	Lunch or snack, transport cost reimbursement for meeting participant			Some on-the-job training provided during routine visits, as required
Implementation period	August 2017– December 2018	May 2016–October 2020	June 2016–July 2017	June 2015–December 2019
Product focus	COC EOC Injectables Implant (2-rod) IUD Male condoms	COC Injectables Implant (2-rod) IUD Male condoms	COC Injectables Implant (1-rod) Implant (2-rod)	COC EOC Injectables IUD Male condoms

Abbreviations: COC, combined oral contraceptives; EOC, emergency oral contraceptives; IMPACT, Information Mobilized for Performance Analysis and Continuous Transformation; IUD, intrauterine device.

a No capacity building in Guinea. In Myanmar, technical advisor supported for first 3 routine meetings.

Four countries implemented IMPACT Teams to improve reproductive health supply chains.

## METHODS

### Quantitative

To examine IMPACT Teams' effect on supply chain outcomes, the study design retrospectively employed a pre-post intervention comparison panel design with a nonrandomized matched comparison group. In Guinea, Indonesia, and Myanmar, the unit of analysis was health facilities, whereas, in Kenya, data were analyzed at the ward level, as facility-level data were not available for all indicators. A ward in Kenya is an administrative structure in a subcounty that reflects electoral assembly units within the devolved county government. A subcounty encompasses several wards, which encompasses several facilities.

Before analysis, all facilities/wards covered by the intervention were matched with comparison facilities/wards using propensity score matching. Comparison facilities/wards in the sampling frame included those not receiving the intervention, those located within the same province or region as intervention facilities/wards, and those that had an entry in the logistics management information system (LMIS) at baseline and endline to allow for the panel design. First, the propensity score or the probability of the facility/ward to be in the intervention area is estimated with a logit model using baseline values of the relevant data available in the country's LMIS as predictors (i.e., beginning stock balance, quantity received, quantity issued, positive and negative adjustments, stock-outs, and average monthly consumption).

Facilities/wards were matched one-to-many, meaning that the propensity scores of facilities/wards from the comparison sampling frame that matched within ±5% of the propensity score of an intervention facility/ward were retained as comparison units and included in the analysis. To assess the adequacy of the matching, t-tests were performed to ensure that the predictors of the final logit model were not statistically significantly different (*P*>.1) between the intervention and the comparison areas. Table 2 presents the number of selected facilities/wards per country and study arm. Given the study's panel design, the same facilities/wards were examined at baseline and endline. No project support was provided to comparison facilities or areas in any of the four countries.

**TABLE 2.** Facilities/Wards Included in a Study to Examine IMPACT Teams Effect on Supply Chain, for Intervention and Matched Comparison Sites

	Guinea	Indonesia	Kenya	Myanmar
No. IMPACT Teams	6	11	10	21
Intervention units: No. facilities/wards covered by IMPACT Teams	67	525	338	489
Comparison units: No. matched comparison facilities/wards	216	1,977	719	903

#### Variables

This analysis focuses on 2 indicators that measure supply chain performance—products stocked according to plan and stock-outs. We selected these indicators because IMPACT Teams in all 4 countries consistently monitored stock levels and stock-out rates for each tracer product as part of their key performance indicator packages, and explicitly aimed to see improvements in both indicators as a result of their work. Other stock-out indicators, such as duration of stock-out, could not be assessed due to the lack of data in national systems. Of note, reporting rates, an indication of data completeness, were consistently high across all intervention and comparison sites.

Stocked according to plan was defined as having a product's calculated months of stock falling between minimum and maximum levels according to national guidelines. This measurement is used to assess inventory management practices and is an important process indicator to predict stock-outs (if months of stock are below the minimum level) or expiries (if months of stock are above the maximum level). A stock-out was defined as having zero usable stock according to the ending balance for each reporting period. Each country's IMPACT Team examined these 2 variables for each tracer product. The tracer products selected represent the most used methods according to each country's method mix (Table 1).

#### Analytic Methods

The analysis used a difference-in-difference fixed effects model to estimate the intervention effect, accounting for matched facilities/wards. The fixed effects model enabled control of unobserved confounders that remain unchanged during the observation period. The difference-in-difference approach enabled the calculation of the intervention's effect on our outcomes of interest (stocked according to plan, stock-outs) by comparing the absolute average change over time for the intervention group, compared to the comparison group. Data were analyzed using Stata 15.1.

### Qualitative

In Kenya and Indonesia, we used key informant interviews to explore barriers and facilitators to IMPACT Team's success and sustainability (Supplement). In Kenya, we reanalyzed a qualitative analysis that was conducted in April 2018 by an independent consultant as part of the project closeout. The initial analysis included 15 key informant interviews conducted using a semistructured interview guide in 5 of the 10 intervention counties, with participation by country team members who played a key role in IMPACT Team implementation. Interviews were recorded and transcribed at the time of the initial study. The research team for this study recoded and reanalyzed the original 2018 transcripts for this analysis, as the research questions for both studies were similar.

In Indonesia, key informant interviews were conducted with 16 government staff, all with significant roles in IMPACT Team implementation. Due to time and resource constraints, respondent selection was prioritized based on availability, the highest level of knowledge of the intervention, and the highest level of engagement at the meetings, according to the implementing partner. Data were collected over the phone in October 2018 by the project focal points in-country who supported IMPACT Teams, using the Kenyan semistructured guide as a base but adapted to address the key questions of this evaluation. The interview guide was structured around the key elements of IMPACT Teams (data, technology, processes, and people), with probes to elicit information on enablers and barriers to successful IMPACT Teams. The guide also addressed issues around IMPACT Team sustainability. Funds were not available to collect qualitative data from Guinea and Myanmar.

All interviews in Kenya were conducted in English; interviews in Indonesia were conducted in Indonesian. Interviews were audio-recorded and subsequently transcribed, and for Indonesia, translated into English for analysis. The qualitative data were coded and analyzed using Nvivo 12 Plus. The initial codebook was developed based on the research themes and questions, as well as researchers' prior knowledge of the intervention. The codebook was further refined as the authors independently examined a sample of interview transcripts to identify emergent themes and finalize the code definitions. The coding results were then synthesized and key findings described and presented back to the Kenyan and Indonesian field teams for validation.

### Ethical Approval

An institutional review board at John Snow, Inc. determined that this activity was exempt from human subject oversight.

## RESULTS

Stock-outs were calculated by taking the percentage of the facilities (or wards in Kenya) with at least one stock-out according to the ending balance of the inventory control cards during a 3-month period. We did not account for multiple stock-outs or the duration of stock-outs.

The percentages presented in the following section are all adjusted rates (Tables 3 and 4). Statistical significance was set at a *P*<.05%.

**TABLE 3.** Adjusted and Statistically Significant Intervention Effects for Supply Chain Stock-Outs

	Intervention Facilities (Adjusted %)			Matched Comparison Facilities (Adjusted %)			Intervention Effect (%)	*P* Value^a^	95% Confidence Intervals
	Pre	Post	Difference	Pre	Post	Difference			
Guinea	N=67			N=216					
COC	39.9	14.3	-25.6	38.9	32.4	−6.5	−19.0	.003	−31.7, −6.3
EOC	46.2	9.2	−37.0	45.8	28.7	−17.1	−19.8	.002	−32.2, −7.4
Injectable	49.3	3.8	−45.5	49.4	23.5	−25.9	−19.6	<.001	−30.3, −8.9
Implant (2-rod)	45.9	10.6	−35.3	43.4	32.7	−10.7	−24.6	.001	−38.4, −10.7
IUD	46.8	8.7	−38.1	42.5	36.0	−6.5	−31.6	<.001	−44.9, −18.3
Male condom	48.7	3.1	−45.6	42.3	32.6	−9.8	−35.8	<.001	−49.7, −21.9
Indonesia	N=525			N=1,977					
COC	41.3	10.7	−30.7	48.7	24.3	−24.4	−6.2	.007	−10.7, −1.7
Injectable	38.6	15.0	−23.6	42.3	26.6	−15.7	−7.9	.001	−12.4, −3.4
Implant (2-rod)^b^	51.6	57.1	5.4	49.5	56.5	7.0	−1.6	.65	−8.5, 5.3
IUD	58.1	45.4	−12.7	56.6	57.4	0.8	−13.5	<.001	−20.6, −6.3
Male condom	52.0	16.5	−35.5	55.4	38.2	−17.2	−18.3	<.001	−22.7, −14.0
Kenya (ward level)	N=338			N=719					
COC	69.6	90.2	20.6	64.6	90.1	25.5	−4.9	.024	−9.1, −0.7
Injectable	65.5	68.8	3.2	58.5	80.4	21.9	−18.6	<.001	−27.5, −9.7
Implant (1-rod)	67.2	59.4	−7.8	62.2	77.3	15.1	−22.8	<.001	−31.6, −14.1
Implant (2-rod)	62.6	36.6	−26.1	61.5	56.9	−4.6	−21.4	<.001	−28.7, −14.2
Myanmar	N=489			N=903					
COC	46.1	5.3	−40.8	59.0	14.1	−44.9	4.1	<.001	2.4, 5.8
EOC	33.6	9.2	−24.4	39.7	8.2	−31.5	7.1	<.001	3.7, 10.4
Injectable	47.9	4.6	−43.2	55.2	20.8	−34.5	−8.8	<.001	−11.7, −5.8
IUD	57.6	5.8	−51.8	34.8	64.8	−29.9	−81.8	<.001	−86.6, −76.9
Male condom	42.4	0.7	−41.7	44.3	4.7	−39.6	−2.1	.003	−3.5, −0.7

Abbreviations: COC, combined oral contraceptives; EOC, emergency oral contraceptives; IUD, intrauterine device.

a Statistical significance is considered at *P*<.05.

b Not statistically significant.

**TABLE 4.** Adjusted and Statistically Significant Intervention Effects for Supply Chain Stocked According to Plan

	Intervention Facilities (Adjusted %)			Matched Comparison Facilities (Adjusted %)			Intervention Effect (%)	*P* Value^a^	95% Confidence Intervals
	*Pre*	*Post*	*Diff*	*Pre*	*Post*	*Diff*			
Guinea	N=216			N=67					
COC	53.3	60.8	7.5	55.0	48.5	−6.5	14.0	.342^b^	−14.9, 42.9
EOC	51.6	75.4	23.8	50.3	65.3	15.0	8.8	.624^b^	−26.4, 43.9
Injectable	51.6	69.3	17.8	50.7	60.0	9.3	8.5	.458^b^	−13.9, 30.9
Implant (2-rod)	53.1	72.2	19.2	56.2	46.0	−10.2	29.4	.032	2.5, 56.2
IUD	51.9	88.7	36.7	56.1	54.6	−1.5	38.2	.046	0.6, 75.8
Male condom	49.9	69.5	19.6	52.8	46.8	−6.0	25.6	.051^b^	−0.1, 51.2
Indonesia	N=525			N=1,977					
COC	50.4	62.7	12.2	51.4	52.6	1.2	11.0	<.001	4.8, 17.2
Injectable	53.1	61.9	8.8	53.5	55.0	1.5	7.3	.007	2.0, 12.6
Implant (2-rod)	49.4	57.0	7.5	50.3	50.4	0.0	7.5	.023	1.0, 13.9
IUD	51.1	58.1	7.1	50.4	55.3	4.7	2.2	.525^b^	−4.5, 9.0
Male condom	49.0	64.6	15.6	48.2	56.0	7.8	7.8	.017	1.4, 14.3
Kenya (ward level)	N=338			N=719					
COC	42.6	29.3	−13.3	46.7	27.3	−19.4	6.1	.060^b^	−0.3, 12.5
Injectable	41.8	33.7	−8.1	43.3	34.3	−9.0	0.9	.801^b^	−5.8, 7.5
Implant (1-rod)	44.6	52.7	8.1	48.1	41.5	−6.6	14.7	<.001	8.3, 21.2
Implant (2-rod)	43.9	59.3	15.4	45.9	48.1	2.3	13.2	<.001	6.0, 20.4
Myanmar	N=489			N=903					
COC	49.8	93.4	43.6	52.4	87.6	35.2	8.4	<.001	4.3, 12.6
EOC	71.6	85.7	14.1	60.0	96.1	36.1	−22.0	.001	−35.6, −8.4
Injectable	51.3	94.0	42.7	51.3	84.8	33.5	9.2	<.001	5.0, 13.4
IUD	48.7	89.1	40.4	72.0	49.9	−22.1	62.5	<.001	35.8, 89.1
Male condom	53.3	96.0	42.7	59.5	94.8	35.3	7.4	.001	3.1, 11.7

Abbreviations: COC, combined oral contraceptives; EOC, emergency oral contraceptives; IUD, intrauterine device.

a Statistical significance is considered at *P*<.05.

b Not statistically significant.

**FIGURE 2:**
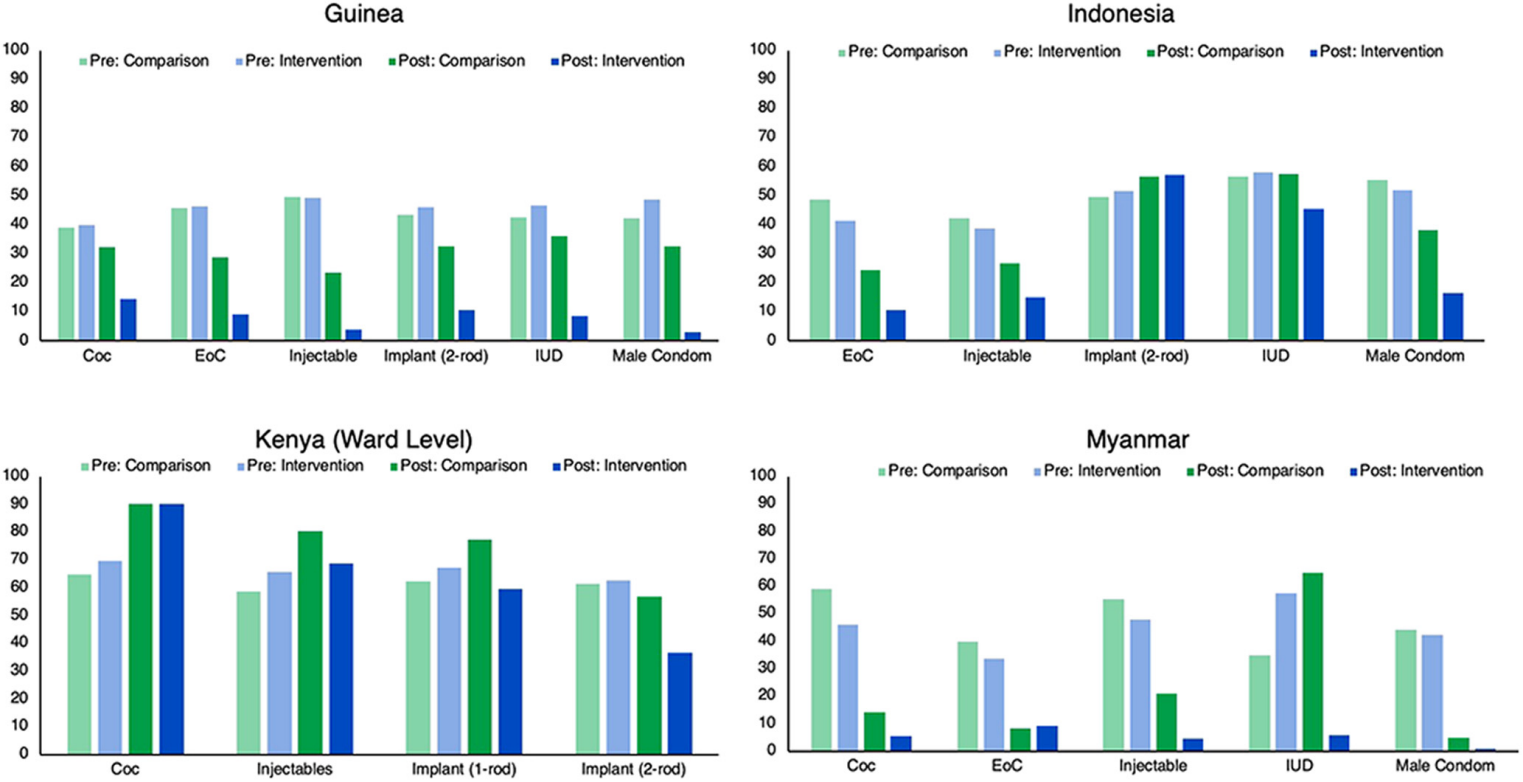
Instances of Stock-Outs Pre- and Post-Data Use Teams for Intervention and Comparison Facilities/Wards, by Country, Adjusted Percentages Abbreviations: COC, combined oral contraceptive; EOC, emergency oral contraceptive; IUD, intrauterine device.

**FIGURE 3:**
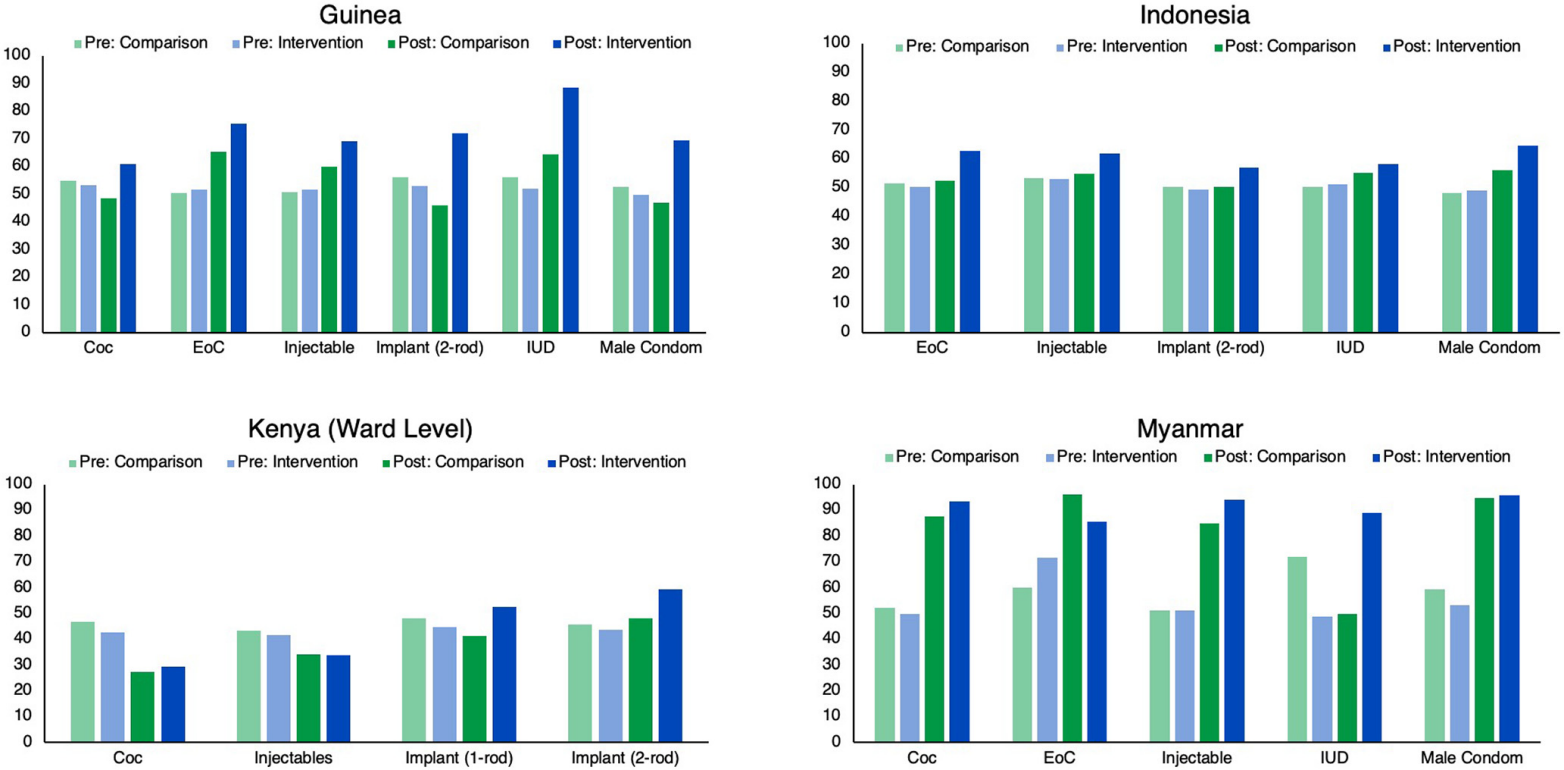
Instances of Products Being Stocked Within Minimum/Maximum Levels Pre- and Post-Data Use Teams for Intervention and Comparison Facilities/Wards, by Country, Adjusted Percentages Abbreviations: COC, combined oral contraceptive; EOC, emergency oral contraceptive; IUD, intrauterine device.

Pre-intervention stock-out and stocked according to plan were very similar for intervention and matched comparison facilities in all countries included in this study. Stock-outs ranged from 34% to 70%, while stocked according to plan ranged from 42% to 72%. With a few exceptions, stock-outs for nearly all products decreased, and stocked according to plan rates increased post-intervention, with statistically significant effects from the intervention, demonstrating the positive effect of IMPACT Teams (Figures 2 and 3).

Stock-outs for nearly all products decreased and stocked according to plan rates increased post-intervention, with statistically significant effects from the intervention.

In Guinea, stock-outs for all products in intervention facilities decreased by 26–46 percentage points, whereas matched comparison sites saw more modest decreases, ranging from only 7–26 percentage points. Similarly, stock-outs in Indonesia decreased by 13–36 percentage points in intervention areas, whereas matched comparison areas had more modest results, including a slight increase in stock-outs for IUDs. Though instances of stock-outs in Kenya increased for combined oral contraceptives (COCs) and injectable contraceptives in both study arms, stock-outs in intervention areas were below the secular trend. For 1-rod implants, intervention wards saw an 8-percentage-point reduction in stock-outs, while stock-outs in matched comparison wards increased by 15 percentage points. More than a third of the intervention period in Kenya was characterized by industrial action (strikes) by nurses and doctors, affecting routine submission of reports and leading to widespread stock-outs across the country. Lastly, in Myanmar, stock-outs declined for all products in both intervention and matched comparison facilities by 24–52 percentage points. Though all intervention effects were significant, COC and emergency oral contraceptive (EOC) stock-outs decreased at a significantly slower rate in intervention facilities than in comparison facilities. This may be a result of other supply chain interventions happening in those areas, including the installment of field officers to monitor and support supply chain activities across health facilities and leadership training provided to state and township staff. The project did not provide any support to comparison units. However, in Southern Shan State, government leadership repeated the IMPACT Team model to townships where the project was not working, and this may have also contributed to positive results in comparison facilities.

In Guinea, the intervention's effect on stocked according to plan was only significant for 2-rod implants and IUDs. However, the trend across all products in intervention facilities moved in a positive direction. In Indonesia, all products, except for IUDs, had a positive intervention effect. In Kenya, significant intervention effects were seen for 1- and 2-rod implants, demonstrating positive impacts from IMPACT Teams. In Myanmar, all intervention effects were statistically significant; the analysis showed that the IMPACT Teams had a positive effect on stock status, except for EOCs, where the intervention effect was negative.

In Kenya and Indonesia, positive supply chain outcomes are attributed to having the people and process components of IMPACT Teams in place, supported by a foundation of strong data and data visualizations. By design, IMPACT Team membership is multidisciplinary, engaging professionals across supply chain functions, health programs, and supply chain levels. Interview respondents in Indonesia and Kenya indicated that this diverse membership led to a more holistic approach to solving supply chain challenges by facilitating collaboration and communication across disciplines and filling in gaps in skills.

*We did not apply this just to family planning commodities. It assisted us…in all other indicators like skilled delivery, fourth ANC, first ANC, PMTCT; we assess data…We know whether we are moving up or moving down. So this knowledge from [IMPACT Teams] has assisted us in all other fields.* —Kenya, RH Coordinator

Respondents appear to be motivated to participate in IMPACT Teams, at least initially, by a sense of professional satisfaction—a feeling that they were able to do their job better, and from a sense of team spirit, as IMPACT Team members work toward a common goal or purpose. Respondents sensed that being part of the team was a driver for accountability in terms of following up on action items assigned to each member. Respondents also mentioned other motivations for continuing as IMPACT Team members, such as access to trainings on new tools for data analysis, making it easier to do one's work; and healthy competition among subunits within a given county/district.

*[A] motivation is the desire to solve problems, because if not, that problem will continue to appear every month. I think it will then create problems in each person's task. So with this QIT we work toward a common goal, which is to lighten the load in each of our jobs.* — Indonesia

Interviewees viewed the IMPACT Team process as a major improvement on the way things were normally done: facilitating better organization in decision making and problem solving and driving action on proposed solutions. Respondents cited standard IMPACT Team processes described in the standard operating procedures as drivers of success, particularly when coupled with usable data and data visualizations.

*Initially, we used to just “solve problems” but now you are forced to go deeper to find out whether the solutions are going to address the real issues.* —Kenya, County Pharmacist

Almost all respondents noted that data framed the discussions about reporting rates and stock levels and served as the basis for sharing requests for support from higher levels of the supply chain. In Indonesia, several interviewees noted that before IMPACT Teams were formed, stocks were allocated evenly across facilities, without consideration for variance in consumption. However, the IMPACT Team approach provided evidence to team members that following the standard procedures improved product availability, which in turn motivated them to follow IMPACT procedures themselves, and to ensure that others in the system did so as well.

*Initially, we would have raw data and try to make sense of it. But now with the IMPACT Team, we came up with tools, the dashboards that now make it easier for us using color coding and graphs. At a glance, you can be able to make decisions quite easily unlike in the past where we used to have a table, try to make sense out of it, trying to draw some conclusions*. —Kenya, County Pharmacist

*In the past, when I planned for distribution in the FP division, it wasn't done based on the [decision support tool]. We basically just look at the quantity of product we have and divide them with the number of SDPs. Now we adjust it to the SDP's need for the next 4 months and we can get the information from that Tool. When the Tool is empty we'll already know which SDPs have a stock-out, and which have an overstock. We'll already know that.* —Indonesia

IMPACT Team approach provided evidence to team members in Indonesia that following the standard procedures improved product availability.

Respondents also frequently mentioned data availability as an opportunity for IMPACT Teams to systematically assess the results of their work by measuring progress against targets. They mentioned the decision-support tools (or dashboards) that visualized the supply chain data as particularly useful for pinpointing problems and comparing changes over time. Additionally, systematically and routinely reviewing data seems to positively impact members' skills in data analysis and data literacy and, in some instances, to improve data quality.

*[IMPACT Team meetings] have helped us to identify some of the data quality gaps like inconsistencies between the closing balance of a particular month and the opening balance of the next month.* —Kenya, Health Information Records Officer

*Data quality is for sure better because we have meetings, so we can discuss the data.* —Indonesia

Despite improvements in supply chain outcomes, Kenya and Indonesia did not see positive improvements in government leadership of IMPACT Team implementation nor a sustained data use culture. The major barrier to IMPACT Teams' sustainability was a lack of government ownership of the IMPACT Team approach. Respondents in both Kenya and Indonesia cited competing priorities, inconsistent attendance, frequent staff transfers, limited participation from health system leaders, and limited human resources as major barriers. Commitment and participation from higher government levels were particular barriers. When leaders sent replacements to participate in IMPACT Team meetings, the substitutes often lacked the understanding of the process necessary to participate properly in meetings and relay information to absent team members.

In Indonesia and Kenya, the major barrier to IMPACT Teams' sustainability was a lack of government ownership of the IMPACT Team approach.

Without support and commitment from higher-level leadership, it is less likely that IMPACT Teams will be able to follow meeting processes, resolve systemic issues, or make decisions, leading members to feel a lack of agency in overcoming supply chain challenges.

*Sometimes in previous meetings the leader is more often present than not. If he's not present, we wait for the decision.* —Indonesia

*What's not enjoyable is when we hope to solve a problem but then it turns out there is no solution for the problem. Procurement of contraceptives, that's something that is outside our ability, and we don't have a solution for it.* —Indonesia

Because governments have not yet taken ownership of the IMPACT Team approach, team members still rely on implementing partners for financial, logistical, and technical support. We found that this was particularly the case when it came to decision-support tools, as there are notable gaps in data analytics and visualization capacity.

*There is no support we are given by the [Ministry of Health] apart from probably getting a conference room to hold the meeting. But on logistics, nothing was supported because you know when we try to initiate that the government says they have no budget for us. It is [the implementing partner] that was supporting us on that end. There is no allocation for the IMPACT Team from the county.* —Kenya, Health Information Records Officer

*Personally the only challenge is that the tool only monitors FP and vaccines and I still have to depend on [the implementing partner] to get data for me.* —Kenya, Chief Pharmacist

It appears that the IMPACT Teams at local levels need support from higher levels to continue their work. At the time of the survey in Indonesia, the implementing partner was still providing mentorship support and technical advice, though respondents did note that some subnational Ministry of Health (MOH) offices had begun including IMPACT Teams as a line item in their budgets, something that merits further exploration. However, in Kenya, where partner support had ended, no counties had independently and consistently continued IMPACT Team meetings.

*Now the team is not that active. It has slowed down its activities. It is now becoming inactive. The coordination of the meeting was mainly being done by [the implementing partner]. They would remind us on when we would have the next meeting and what the agenda would be. The steering committee is now not that active. That is what is happening* —Kenya, Health Information Records Officer

*If [the implementing partner] leave us, is there a technique that can help enable people to do this on their own? … This mentorship has been crucial in my opinion. I know this is an activity that I cannot do on my own, everyone is busy.* —Indonesia

## DISCUSSION

Our results across all 4 countries demonstrate that IMPACT teams are an effective approach for improving contraceptive supply chain inventory management and commodity availability, at least in the short term. Our analysis revealed significant positive changes in stock status and stock-out rates across most products. For nearly all products across the 4 countries, the increase in products stocked according to plan and the reduction in stock-outs can be attributed to the IMPACT Team intervention. Stock-outs improved more consistently than stock status and this may be attributed to the fact that stock-outs are easier to fix at the facility level by moving stock around. This may result in understocking across facilities therefore not improving stock status but preventing stock-outs. Achieving stock status across all facilities requires adequate and reliable stock from the national level and this cannot always be fixed by the IMPACT teams and points to the need for effective leadership.

For nearly all products across the 4 countries, the increase in products stocked according to plan and the reduction in stock-outs can be attributed to the IMPACT Team intervention.

Despite these successes in strengthening supply chain performance in the short term, our efforts did not lead to the establishment of government leadership and ownership necessary to sustain a data use culture and build stronger systems. Due to data limitations, we could not fully examine and demonstrate a clear causal pathway as depicted in our theory of change, namely, that government leadership leads to the installation of a data use culture, which in turn leads to improved product availability. Rather, we believe that a data use culture—rather than product availability—is the outcome of interest for sustained change.

We believe that a data use culture—rather than product availability—is the outcome of interest for sustained change.

IMPACT Teams originated as a continuous quality improvement approach, with the premise that teams routinely review data, problem solve, and plan and undertake specific, responsive actions to address the challenges implied by the data and identified during the root cause analysis process. To sustain both the data use culture and the improved outcomes (which were achieved by continuous actions to solve challenges identified by the indicators), teams must hold routine meetings to undertake the data review practice and cycle, and managers at all levels of the system must use data as a way to understand problems and take actions. Thus, it is important for government leadership to signal the importance of using this approach to improve supply chain performance by creating an enabling environment for IMPACT team meetings and modeling the practice of data use for problem solving.

Many respondents noted the key role that implementing partners have played in IMPACT Teams. They expressed mixed opinions on whether the MOHs will be able to sustain these teams once projects end. Interviewees mentioned such barriers as insufficient financial support and inadequate, although improving, data analysis and data literacy skill levels, which have left many cautious about the feasibility of post-intervention implementation. In Indonesia, some subnational MOH offices had budgeted for IMPACT Teams; but, in Kenya, IMPACT Teams no longer met consistently once partner support ended.

While IMPACT team members appreciated the value and benefits of the approach, to ensure embedded MOH ownership and drive toward broader uptake, we hypothesize that several factors needed to be institutionalized to achieve sustained data use. First, a longer implementation period would enable the effectiveness of the approach to be documented and disseminated with the involvement of IMPACT team members. Second, operationalization of the IMPACT teams at multiple levels, especially at the national level, would enable policy makers to appreciate the benefits directly and begin to adopt data and evidence as a basis for actions and decisions. Third, ensuring that support could be sufficiently planned and incorporated into national or district budgets may also have enabled institutionalization of the approach over time because financial constraints were an important limitation (Box 2).

BOX 2 **Unexpected Insights That Provide Meaningful Implications for Future Data Use Initiatives**

**Information Mobilized for Performance Analysis and Continuous Transformation** (**IMPACT) Teams at the district/subcounty level were most effective in meeting and creating targeted action plans.** They were close enough to the problem to understand the facility-level challenges necessary for a root cause analysis, serve an administrative role, and are empowered to make changes or elevate more complex issues to higher levels.

*That is why we cascaded to the sub-county level, where they can address those challenges there and put in measures to improve on … They are able to initiate their own methods from the facility level and subcounty level and ensure that the improvement is done. That is the outcome you get from them and take to the county.* —Kenya, Health Information Records Officer

In theory, IMPACT Teams are supposed to be interconnected by level, enabling lower-level teams to escalate problems that are beyond their purview to solve to teams at higher levels, which have the authority to address such problems. However, no national IMPACT Team was established in Kenya or Guinea. In Myanmar, 2 states established a state-level IMPACT Team, which was useful in motivating lower-level teams, but no national team was initiated. The challenges of initiating IMPACT Teams at the national level suggest that the approach might have to be redesigned in terms of meeting structure, processes, and identifying different motivations for national-level stakeholders to coordinate effectively and regularly on a cohesive agenda.

Despite this lack of high-level leadership, **IMPACT Teams have developed and implemented innovative, locally driven solutions beyond standard supply chain activities**. Examples include redistributing reporting tools, instituting peer-to-peer capacity building on stock management and ordering, overhauling a dysfunctional filing system at the district level, creating a panel responsible for increasing reporting rates and improving data quality, creating technical working groups within IMPACT Teams, and using WhatsApp as a mechanism for communicating stock status due dates, and even for reporting data.

*For the service delivery point level, the WhatsApp is also constantly being used, so if any SDP experiences a stock-out, it'll be notified through WhatsApp.* —Indonesia

Based on the findings from this study, IMPACT Teams have taken a very different approach, drawing heavily on human-centered design principles that seek to involve the end users, in this case, the national and subnational MOH, in defining the problem and cocreating solutions. Starting in 2019, IMPACT Teams were redesigned alongside MOH supply chain actors in Kenya and Tanzania, gaining a better understanding of motivations and incentives for adopting IMPACT Teams, as well as gaining a better landscape of the current supply chain operations, to find ways to implement the approach within existing mechanisms. We have also taken a more people-centered approach to IMPACT Teams, understanding that it is not just data availability or partner-supported meetings that will lead to sustained change but rather a holistic set of skills, behaviors, and competencies that are needed to make this approach a lasting one.

Our revised hypothesis is that government leadership and a data use culture are important for scaling, institutionalizing, and sustaining gains in supply chain outputs such as product availa-bility,^21^ and that not truly understanding the motivations and incentives of leadership and team member participation was one of the main reasons for stops and starts in gains achieved and the lack of long-lasting change.

### Limitations

As a retrospective study, this study had limitations. As projects implemented activities using an integrated approach, it was not possible to isolate the effect of IMPACT Teams from the intervention package as a whole. Therefore, any reference to IMPACT Teams or to the intervention in a particular country in this article reflects the complete set of activities conducted in that country. However, IMPACT teams were the common intervention across the countries, and IMPACT teams were clearly a contributing factor to the positive results. Additionally, data comes from the LMIS; and during implementation, countries were also working on strengthening their LMIS systems. Therefore, some improved indicators might in part be due to more complete and accurate data. As this was a retrospective study, there were no true control areas. It is possible that in the matched comparison areas at least some type of supply chain support was being provided by a partner, but the projects were not fully aware of what type of support was being provided and this could not be factored into the interpretation of results. Lastly, not enough research was conducted to understand the reasons behind low leadership motivation and engagement, which, if done during the course of implementation, might have enabled course corrections towards sustainability of IMPACT teams.

## CONCLUSION

IMPACT Teams have proven to be effective in reducing stock-outs and increasing adequate stock levels. However, there is still work to be done in understanding how to truly create a culture of data use and ensure the sustainability of the approach. The core components of data, technology, process, and people that are rooted in the quality improvement approach need to be accompanied by specific activities to foster government ownership and leadership so that data use processes are not only instilled but sustained over time. In addition, plans for future supply chain data use initiatives should cover how IMPACT Teams can be adapted to different contexts, such as the level of data literacy skills, leadership buy-in, and resources available. Redesigning the IMPACT Team approach using human-centered design to be more adaptable to context and user needs and barriers may be a way to increase the motivation needed for sustained involvement at all levels of the supply chain.

## Supplementary Material

GHSP-D-21-00345-supplement.pdf

## Translation

**En Français**

**Des données pour action : une étude par méthodes mixtes portant sur les équipes d'utilisation des données, l'amélioration de la disponibilité des contraceptifs et le rôle de la gouvernance en Guinée, en Indonésie, au Kenya et au Myanmar**

**RÉSUMÉ**

Les équipes IMPACT (Information Mobilized for Performance Analysis and Continuous Transformation – Information mobilisée pour l'analyse des performances et la transformation continue) utilisent une approche centrée sur les personnes et axée sur les données pour renforcer les chaînes d'approvisionnement en favorisant un cycle continu d'amélioration de la chaîne d'approvisionnement. Les équipes IMPACT rassemblent régulièrement des données, des personnes, des processus et des technologies, sous la direction du gouvernement, pour instaurer un changement de culture qui conduit à des améliorations durables des processus et des résultats de la chaîne d'approvisionnement. Cette étude par méthodes mixtes a cherché à savoir si les équipes IMPACT étaient efficaces pour améliorer les résultats de la chaîne d'approvisionnement de la santé de la reproduction en Guinée, en Indonésie, au Kenya et au Myanmar, et a identifié les facteurs favorables et les obstacles au succès et à la pérennisation des équipes IMPACT en Indonésie et au Kenya.

La conception de l'étude s'est appuyée sur une conception de panel de comparaison avant et après intervention avec un groupe témoin apparié non aléatoire pour examiner l'effet des équipes IMPACT sur 2 résultats de la chaîne d'approvisionnement : ayant un stock adéquat, et ayant des ruptures de stock. Des entretiens supplémentaires avec des informateurs clés menés au Kenya et en Indonésie ont permis d'explorer les obstacles et les facteurs favorables du succès et de la pérennisation des équipes IMPACT.

Pour la plupart des produits dans les quatre pays, une augmentation des produits ayant un stock adéquat et une réduction des ruptures de stock peuvent être attribuées à l'intervention de l'équipe IMPACT, ce qui démontre que les équipes IMPACT représentent une approche efficace pour améliorer la gestion et la disponibilité des stocks de la chaîne d'approvisionnement des contraceptifs. Toutefois, nos résultats ne révèlent pas un lien de causalité clair, tel que théorisé dans notre Théorie du changement (TdC), à savoir que le leadership du gouvernement conduit à l'installation d'une culture d'utilisation des données, qui à son tour conduit à une meilleure disponibilité des produits. Au Kenya et en Indonésie, bien que la disponibilité des produits se soit améliorée, il y a eu un manque de leadership et de changement de culture. Cela porte à croire que l'amélioration de la disponibilité des produits ne dépend pas de l'établissement d'une culture d'utilisation des données ou du leadership du gouvernement, mais plutôt d'une culture d'utilisation des données – et que la disponibilité des produits - est le résultat d'intérêt pour le changement pérenne, et que la compréhension des motivations et des incitations à la participation du leadership peut être plus importante pour la mise à l'échelle, l'institutionnalisation et le maintien des acquis dans les résultats de la chaîne d'approvisionnement.

**Message clé de l'approche**

Les équipes IMPACT utilisent une approche centrée sur les personnes et axées sur les données pour renforcer les chaînes d'approvisionnement en favorisant un cycle continu d'amélioration de la chaîne d'approvisionnement. Cette étude démontre que les équipes IMPACT représentent une approche efficace pour améliorer la gestion et la disponibilité des stocks de contraceptifs dans la chaîne d'approvisionnement, mais que des recherches supplémentaires sont nécessaires pour comprendre les motivations des dirigeants et les incitations à la participation, qui sont susceptibles de conduire à l'établissement d'une culture d'utilisation des données et sont essentielles pour la pérennisation.

**MESSAGES CLÉS**

- Les équipes d'utilisation des données (IMPACT) représentent une stratégie efficace pour améliorer les résultats de la chaîne d'approvisionnement en santé de la reproduction à court terme, mais elles ne peuvent être maintenues que par l'appropriation et l'engagement des dirigeants.
- Bien que les interventions des équipes IMPACT dans les quatre pays aient conduit à des améliorations de la chaîne d'approvisionnement, l'établissement de la culture d'utilisation des données a été limité.
- Le manque d'engagement des entités gouvernementales de haut niveau - et l'absence de soutien financier en faveur du processus de l'équipe IMPACT - ont constitué des obstacles majeurs à la pérennisation de l'approche de l'équipe IMPACT.

**PRINCIPALES IMPLICATIONS**

- Les composantes principales que sont les données, la technologie, les processus et les personnes, qui sont ancrées dans l'approche d'amélioration de la qualité, doivent être accompagnées d'efforts délibérés pour favoriser l'appropriation et le leadership du gouvernement, afin que ce processus soit non seulement instauré, mais soutenu dans le temps.

**Peer Reviewed**

**First published online:** June 24, 2022.
