# Supplementary material for: Data to Action: A Mixed-Methods Study of Data Use Teams, Improved Availability of Contraceptives in Guinea, Indonesia, Kenya, and Myanmar
Source: Glob Health Sci Pract. 2022 Jun 29;10(3):e2100345. doi: 10.9745/GHSP-D-21-00345 (PMC9242612; doi:10.9745/GHSP-D-21-00345)
Supplement: GHSP-D-21-00345-supplement.pdf [file GHSP-D-21-00345-supplement.pdf]

Supplement to: Yongho A, Chandani Y, Anderson S, Karim A, Saad B, Keddem C. Data to action: a mixed-methods study of data use teams, improved availability of contraceptives and the role of governance in Guinea, Indonesia, Kenya, and Myanmar. *Glob Health Sci Pract*. 2022;10(3):e2100345. https://doi.org/10.9745/GHSP-D-21-00345

Supplement Table. Direct quotations to support evidence of enablers and barriers

	Enablers		Barriers	
	**Key Themes**	**Illustrative Quotes**	**Key Themes**	**Illustrative Quotes**
**People**	• Team members are motivated by having access to tools and gaining capacity to analyze data<br>• Members are motivated by their involvement in something they perceive to be "new" and "innovative" and by their desire for it to succeed<br>• Members are motivated by watching data illustrate improvements & by interacting with other districts to compare successes<br>• Sense of responsibility to the team, shared goals and other team members' enthusiasm is motivating and helps to develop a sense of team spirit<br>• Members are motivated by praise and recognition - being rewarded without financial compensation	*"I think staff are motivated by how we handle them. So long as the administration recognizes their work, and most of the time we give credit to the best performing facility. That gives other facilities changes to pull up." - Kenya, HRIO*<br><br>*"…from the MIM tool, we can see our performance, how we are doing... so we become interested in trying to solve the problem. We are fired up to see that data there. That's the result of our work." - Indonesia*<br><br>*"[A] motivation is the desire to solve problems, because if not, that problem will continue to appear every month. I think it will then create problems in each person's task. So with this QIT we work toward a common goal, which is to lighten the load in each of our jobs." - Indonesia*	• Inconsistent attendance and frequent staff transfers stall momentum<br>• Inconsistent capacity of newly transferred staff is a challenge<br>• Without higher level leadership support and commitment, more systemic issues cannot be resolved, leading to demotivated members<br>• Competing priorities and lack of time hinders meeting attendance<br>• Lack of incentives for participation (transportation assistance, refreshments, compensation) hinders meeting attendance<br>• Leaders are frequently absent and decisions cannot be made if leader is absent	*"We expect everyone to be present at meetings... However, to synchronize everyone's schedule that is extraordinarily difficult." - Indonesia*<br><br>*"A staff who is starting to get a grasp of things, has participated in a few exercises, is willing to go with QIT to the field, suddenly he's transferred. So we're back to zero again." - Indonesia*

Supplement to: Yongho A, Chandani Y, Anderson S, Karim A, Saad B, Keddem C. Data to action: a mixed-methods study of data use teams, improved availability of contraceptives and the role of governance in Guinea, Indonesia, Kenya, and Myanmar. *Glob Health Sci Pract*. 2022;10(3):e2100345. https://doi.org/10.9745/GHSP-D-21-00345

	Enablers		Barriers	
	**Key Themes**	**Illustrative Quotes**	**Key Themes**	**Illustrative Quotes**
**Processes**	• IMPACT Teams facilitate coordination and communication between team members and help fill skill gaps<br>• The process of accountability - following up on tasks assigned to each member at meetings - moves things forward<br>• Process is perceived as fostering innovation and local solutions	*"Initially we used to just 'solve problems' but now you are forced to go deeper to find out whether the solutions are going to address the real issues." - Kenya County Pharmacist*<br><br>*"Whenever we involve the sub-county we are able to initiate the changes from their level. We cannot do everything from the county to the sub-county level. We give them authority to initiate changes and improve on the mandate and roles indicated." - Kenya, HRIO*<br><br>*"We all know that commodity security is very important and if there are no skills then it means that things will not work well, so during these meetings skills gap are addressed, like how to go about calculations of commodities." - Kenya, RH Coordinator*<br><br>*"One of the ways we recommended for us to get those reports and improve the reporting rates, was for them to take a photo and then when they get to a place with network, they can share the photos via Whatsapp. At the headquarters we key in that data for them." - Kenya, County Pharmacist*	• Partner support (financial and technical) still seen as necessary for IMPACT Teams functioning as there is a lack of resources and infrastructure to conduct meetings<br>• Gaps in skills and understanding of data analysis and continuous review cycle inhibits the process<br>• Inability to follow through on actions established at meetings due to external challenges	*"There is no support we are given by the MOH apart from probably getting a conference room to hold the meeting. But on logistics, nothing was supported because you know when we try to initiate that the government says they have no budget for us. It is JSI that was supporting us on that end. There is no allocation for the IMPACT Team from the county." - Kenya, HRIO*<br><br>*"We don't always do them all [standard process]. For example, there are times when we don't do root cause analysis. Sometimes we don't give recognition. We do have meeting minutes, data review, problem solving. Those are always done." - Indonesia*

Supplement to: Yongho A, Chandani Y, Anderson S, Karim A, Saad B, Keddem C. Data to action: a mixed-methods study of data use teams, improved availability of contraceptives and the role of governance in Guinea, Indonesia, Kenya, and Myanmar. *Glob Health Sci Pract*. 2022;10(3):e2100345. https://doi.org/10.9745/GHSP-D-21-00345

	Enablers		Barriers	
	**Key Themes**	**Illustrative Quotes**	**Key Themes**	**Illustrative Quotes**
**Data & Technology**	● Provides a strong basis on which to plan and make decisions<br>● Data visualizations help members understand and pinpoint problems and to compare changes over time<br>● Data quality is a problem but can be addressed through the process of routine review	*"…we were able to project the data and see the data on commodities and the other data on the overall reporting. So it was very nice when we met because were able to bring all the stakeholders' reports. We were able to discuss at a table."*<br>*- Kenya, HRIO*<br><br>*"Initially we would have raw data and try to make sense of it. But now with the IMPACT Team, we came up with tools, the dashboards that now try to make it easier for us using color coding and graphs. At a glance you can be able to make decisions quite easily unlike in the past where we used to have a table, try to make sense out of it, trying to draw some conclusions."*<br>*- Kenya, County Pharmacist*<br><br>*"[IMPACT Team meeting have] helped us to identify some of the data quality gaps like inconsistencies between the closing balance of a particular month and the opening balance of the next month." - Kenya, County Pharmacist*<br><br>*"Data quality is for sure better because we have meetings, so we can discuss the data." - Indonesia*	● Unreliable networks make updating online data challenging<br>● Lack of reporting tools results in incomplete data<br>● Inconsistent data accuracy is a barrier to trusting the data<br>● Reliance on partners to create dashboard and conduct analysis limits sustainability	*"Most of the time the data at the facility level and the DHIS were not corresponding. So there was a poor reporting system. So it took time until they were able to go to the lower division that was when we saw data rhyming." - Kenya, RH Coordinator*<br><br>*"Personally the only challenge is that the tool only monitors FP and vaccines and. I still have to depend on JSI to get data for me." - Kenya, Chief Pharmacist*

Supplement to: Yongho A, Chandani Y, Anderson S, Karim A, Saad B, Keddem C. Data to action: a mixed-methods study of data use teams, improved availability of contraceptives and the role of governance in Guinea, Indonesia, Kenya, and Myanmar. *Glob Health Sci Pract*. 2022;10(3):e2100345. https://doi.org/10.9745/GHSP-D-21-00345

	Enablers		Barriers	
	**Key Themes**	**Illustrative Quotes**	**Key Themes**	**Illustrative Quotes**
**Government Ownership & Leadership**	• Team recommendations have strong influence on leaders' decisions<br>• Upper level interest in the work and using findings to inform decision making was demonstrated in some instances	*"The decisions that the leader makes is based on recommendations from the [IMPACT Team]. So I think the team's influence is extraordinary…" - Indonesia*	• Lack of direction or involvement from government leadership is a challenge<br>• Frequent changes in leadership stalls momentum<br>• Lack of IMPACT Team member influence on higher-level decision making leads to inaction	*"The ones that haven't gone well is like this, after a meeting we've set the monitoring to be a certain date. Sometimes things don't go as planned, since one person is not available, another is on travel, so we can't do it. It's postponed, postponed, postponed." - Indonesia*<br><br>*"What's not enjoyable is when we hope to solve a problem but then it turns out there is no solution for the problem. Procurement of contraceptives, that's something that is outside our ability, and we don't have a solution for it." - Indonesia*
**Insights on Sustainability**	• Formal decrees or other forms of institutionalization of the process (standard SOPs) is key to sustainability<br>• Funding from local or state budget have begun in Indonesia and is seen as a driver of sustainability<br>• Mentorship and refresher training should be instituted to ensure competency of new members	*"Finances are actually not a problem. We've been independent all this time… there's none (transport assistance). It's part of their main task and function." - Indonesia*<br><br>*"After JSI …. It's actually simple to hold a meeting, basically what is available is adequate, the refreshments. The one that is missing is the transport, back then with JSI transport was also provided, but not anymore now, we do not*	• More than financial support, the coordination aspect has been challenging in Kenya once project funds ended<br>• While a potential solution, mentorships is a challenging skill to acquire	*"But now the team is not that active. It has slowed down its activities. It is now becoming inactive. The coordination of the meeting was mainly being done by JSI. They would remind us on when we would have the next meeting and what he agenda would be. The steering committee is now not that active. That is what is happening" - Kenya, HRIO*

Supplement to: Yongho A, Chandani Y, Anderson S, Karim A, Saad B, Keddem C. Data to action: a mixed-methods study of data use teams, improved availability of contraceptives and the role of governance in Guinea, Indonesia, Kenya, and Myanmar. *Glob Health Sci Pract*. 2022;10(3):e2100345. https://doi.org/10.9745/GHSP-D-21-00345

	Enablers		Barriers	
	**Key Themes**	**Illustrative Quotes**	**Key Themes**	**Illustrative Quotes**
	• Buy-in from county level management/ directors/leaders that expect IMPACT Team outputs to be routine is essential<br>• Integrating IMPACT Team with other existing activities may reduce issues of conflicting priorities	*allocate it, we only have refreshments. That is from the local budget" - Indonesia*<br><br>*"Since it's been predicted and planned since the start, JSI has coordinated this, so in 2018 when no more funding is available from JSI [for QITs] we put it in the budget implementation checklist (DIPA)." - Indonesia*<br><br>*"Yes, now you see we are not looking them as the impact team meetings, we are looking at them as a commodity, security, technical working committee." - Kenya, County Pharmacist*		*"If [JSI] leave us, is there a technique that can help enable people to do this on their own? ... This mentorship has been crucial in my opinion. I know this is an activity that I cannot do on my own, everyone is busy". - Indonesia*
